# Early presence of anti-angiogenesis-related adverse events as a potential biomarker of antitumor efficacy in metastatic gastric cancer patients treated with apatinib: a cohort study

**DOI:** 10.1186/s13045-017-0521-0

**Published:** 2017-09-05

**Authors:** Xinyang Liu, Shukui Qin, Zhichao Wang, Jianming Xu, Jianping Xiong, Yuxian Bai, Zhehai Wang, Yan Yang, Guoping Sun, Liwei Wang, Leizhen Zheng, Nong Xu, Ying Cheng, Weijian Guo, Hao Yu, Tianshu Liu, Pagona Lagiou, Jin Li

**Affiliations:** 1 Department of Epidemiology, Harvard T. H. Chan School of Public Health, 677 Huntington Avenue, Boston, MA 02115 USA; 2 Fudan University Zhongshan Hospital, Shanghai, China; 3 People’s Liberation Army Cancer Center, 81st Hospital of People’s Liberation Army, Beijing, China; 4 Academy of Military Medical Sciences, 307th Hospital of PLA, Beijing, China; 5 First Affiliated Hospital of Nanchang University, Nanchang, China; 6 Harbin Medical University Cancer Hospital, Harbin, China; 7 Shandong Cancer Hospital, Jinan, China; 8 Gansu Cancer Hospital, Lanzhou, China; 9 First Affiliated Hospital of Anhui Medical University, Hefei, China; 10 Shanghai First People’s Hospital, Shanghai, China; 11 XinHua Hospital Affiliated to Shanghai Jiaotong University, Shanghai, China; 12 First Affiliated Hospital of Zhejiang University, Hangzhou, China; 13 Jilin Provincial Cancer Hospital, Changchun, China; 14 Fudan University Shanghai Cancer Center, Shanghai, China; 15 Nanjing Medical University, Nanjing, China; 16 Department of Hygiene, Epidemiology and Medical Statistics, School of Medicine, National and Kapodistrian University of Athens, 75 M. Asias Street, Goudi GR, 115 27 Athens, Greece; 17 Department of Oncology, Shanghai East Hospital, Tongji University School of Medicine, No. 150 Ji Mo Road, Shanghai, 200120 People’s Republic of China

**Keywords:** Apatinib, Gastric cancer, Biomarker, Adverse events

## Abstract

**Background:**

Reliable biomarkers of apatinib response in gastric cancer (GC) are lacking. We investigated the association between early presence of common adverse events (AEs) and clinical outcomes in metastatic GC patients.

**Patients and methods:**

We conducted a retrospective cohort study using data on 269 apatinib-treated GC patients in two clinical trials. AEs were assessed at baseline until 28 days after the last dose of apatinib. Clinical outcomes were compared between patients with and without hypertension (HTN), proteinuria, or hand and foot syndrome (HFS) in the first 4 weeks. Time-to-event variables were assessed using Kaplan–Meier methods and Cox proportional hazard regression models. Binary endpoints were assessed using logistic regression models. Landmark analyses were performed as sensitivity analyses. Predictive model was analyzed, and risk scores were calculated to predict overall survival.

**Results:**

Presence of AEs in the first 4 weeks was associated with prolonged median overall survival (169 vs. 103 days, log-rank *p* = 0.0039; adjusted hazard ratio (HR) 0.64, 95% confidence interval [CI] 0.64–0.84, *p* = 0.001), prolonged median progression-free survival (86.5 vs. 62 days, log-rank *p* = 0.0309; adjusted HR 0.69, 95% CI 0.53–0.91, *p* = 0.007), and increased disease control rate (54.67 vs. 32.77%; adjusted odds ratio 2.67, *p* < 0.001). Results remained significant in landmark analyses. The onset of any single AE or any combinations of the AEs were all statistically significantly associated with prolonged OS, except for the presence of proteinuria. An AE-based prediction model and subsequently derived scoring system showed high calibration and discrimination in predicting overall survival.

**Conclusion:**

Presence of HTN, proteinuria, or HFS during the first cycle of apatinib treatment was a viable biomarker of antitumor efficacy in metastatic GC patients.

**Electronic supplementary material:**

The online version of this article (10.1186/s13045-017-0521-0) contains supplementary material, which is available to authorized users.

## Background

Gastric cancer (GC) is the fourth most common cancer and the second leading cause of cancer-related deaths worldwide, with more than 700,000 deaths annually [1]. Although the global incidence of GC is down-trending, Asia is still with the highest incidence. China has almost 42% of the GC cases worldwide. Every year, there are about 679,000 new cases and 498,000 GC related deaths in China [2], which is a heavy burden to public health.

As symptoms of early GC are usually atypical and unnoticed, many patients are diagnosed at an advanced stage accompanied by extensive invasion and lymphatic metastasis, with an overall survival (OS) of 3 to 5 months if left untreated [1, 2]. Although first-line chemotherapy provides a 6-month survival benefit for patients with advanced GC, second-line chemotherapy with irinotecan or docetaxel adds only about 1.5 months to OS [3, 4]. The recent REGARD [5] and RAINBOW [6] trials has led to the approval of ramucirumab (a monoclonal antibody VEGFR-2 antagonist) alone and in combination with paclitaxel, in second-line treatment of GC. Ramucirumab provided a 1.4 months’ benefit alone [5] and a 2.2 months’ OS benefit in addition to paclitaxel [6]. However, there is no standard third-line treatment if second-line chemotherapy fails.

Apatinib, a novel oral small molecule tyrosine kinase inhibitor targeting VEGFR-2, has demonstrated good safety, tolerability, and efficacy in the treatment of patients with advanced metastatic GC based on phase I–III trials [7–9]. Compared with the placebo group, the progression-free survival (PFS) and OS of the apatinib groups were significantly prolonged by around 2 months. Based on the phase II and III trials [8, 9], apatinib was approved by Chinese Food and Drug Administration in advanced GC in 2014.

Apatinib provides a promising treatment for patients who have failed second-line chemotherapy. The investigation into predictive biomarkers of its anti-antiogenic activity is therefore a challenge and gains high priority. In a phase III trial [9], we observed that GC patients with anti-angiogenesis related adverse events (AEs), namely hypertension (HTN), proteinuria, and hand and foot syndrome (HFS), tended to have better clinical outcomes. These AEs are frequently reported in treatments with other angiogenesis inhibitors and have been suggested as surrogates of the anti-angiogenic activity. Similarly, a study of 80 apatinib-treated advanced breast cancer patients showed that both HTN and high expression of p-VEGFR2 could be biomarkers for good treatment efficacy [10].

Based on these observations, we conducted a retrospective cohort study to investigate the association of anti-angiogenesis related AEs with clinical outcomes in metastatic GC patients, using data from phase II and III trials. More specifically, we aimed to investigate whether HTN, proteinuria, and HFS during the first cycle of apatinib treatment could predict longer OS of metastatic GC patients and serve as a biomarker of antitumor efficacy.

## Methods

To investigate the relationship between adverse effects and antitumor efficacy, we pooled data from 269 apatinib-treated metastatic GC patients in the two prospective multicenter clinical trials [8, 9]. One study was a randomized, double-blind, placebo-controlled phase II trial [8] in which 93 patients received oral apatinib 850 mg once daily or 425 mg twice daily. The other study was a randomized, double-blind, placebo-controlled phase III trial [9], and 176 patients in the treatment arm of this study received apatinib 850 mg once daily. One treatment cycle was 28 days long. Treatment interruptions, dose reductions, and supportive care were allowed for the management of AEs.

Eligibility criteria for all patients in the present analyses included age between 18 and 70 (inclusive) years; histologically confirmed advanced GC or metastatic GC (including gastroesophageal junction adenocarcinoma); prior lack of response or intolerance to at least two lines of chemotherapy; at least one measurable lesion as defined by Response Evaluation Criteria in Solid Tumors (RECIST); an Eastern Cooperative Oncology Group performance status (ECOG PS) of 0 or 1; and acceptable hematologic, hepatic, and renal function. Patients with uncontrolled blood pressure on medication (> 140/90 mmHg), those with a bleeding tendency, and those receiving thrombolytics or anticoagulants were excluded.

Tumor assessments were performed based on computed tomography and/or magnetic resonance imaging at baseline, after cycles two and three, and every 8 weeks thereafter until disease progression and were evaluated according to RECIST (version 1.0 [11] in phase II trial and 1.1 [12] in phase III trial). Efficacy measures included OS, PFS, objective response rate (ORR; including rate of complete response plus partial response), and disease control rate (DCR; including complete response, partial response, and stable disease). AEs (classified and graded using the National Cancer Institute Common Terminology Criteria for Adverse Effects version 3.0 [13]) were assessed at baseline until at least 28 days after the last dose of study drug was administered.

The primary exposure was the presence of any of the three AEs (HTN, proteinuria, and HFS) in the first 4 weeks of treatment. The cut-off was chosen at 4 weeks (1 cycle) after initiation of therapy because of high prevalence in the first 4 weeks and clinical relevance that efficacy measurements and change of treatment plans usually occur according to cycles. Other exposures of interest included different times of onset and different combinations of AEs. Baseline characteristics were compared using *t* test, Wilcoxon rank sum test, chi-square test, and Fisher exact test.

The primary outcome was OS, defined as time from random assignment to death or withdrawal or end of study, whichever occurred first. Secondary outcomes included PFS, DCR, and ORR. Time-to-event endpoints were assessed using Kaplan–Meier methods and compared between groups using the log-rank test. Cox proportional hazard regression models were used to estimate hazard ratios (HR). Proportional hazard assumption was assessed by including the exposure as a time-dependent covariate. Binary endpoints were assessed using logistic regression models. Potential confounders including age, sex, ECOG PS, and number of metastatic sites were adjusted in multivariable regression models. Effect modification by age, sex, and ECOG PS were tested by including interaction terms in the analyses.

To avoid the bias caused by the time-dependent definition of exposures of interest, landmark analyses were performed by excluding subjects who died or had disease progression or death before the landmark (set at 1 month after initiation of apatinib therapy) from the OS and PFS analyses, respectively, as sensitivity analyses.

A predictive model was developed using Cox proportional hazards, with each factor investigated in univariate and then multivariate analyses with a forward stepwise algorithm. Factors in the univariate analysis with a *P* value of less than 0.01 were entered into the multivariate model. Risk scores were calculated according to the model. Calibration and discrimination were evaluated [14].

Missing values were handled using complete case analysis for exposure and outcomes and available case analysis for other covariates. Two-sided tests were used, and a *P* value < 0.05 was considered statistically significant. All statistical analyses were carried out with Stata 14.0, R 3.0 and Revman 5.

## Results

Demographics and baseline characteristics of patients included in this analysis are presented in Table 1. A total of 269 patients with metastatic GC were included in the pooled analysis. The median OS was 139 days (interquartile range, 82–236 days), and the median PFS was 78 days (interquartile range, 54–143 days). By the end of the study, 231 (85.9%) patients had progressed and 209 (77.7%) had died. The overall DCR was 44.98% and ORR was 6.32%.

**Table 1:** Characteristics of 269 apatinib-treated gastric cancer patients from two clinical trials included in the present study

	Without adverse events^a^ No. (%)	With adverse events No. (%)	*P* value^b^
Overall	119 (44.24)	150 (55.76)	
Trial			
Phase II	45 (37.82)	48 (32.00)	0.319
Phase III	74 (62.18)	102 (68.00)	
Age (years)			
< 30	2 (1.68)	1 (0.67)	0.319
30–39	10 (8.40)	8 (5.33)	
40–49	23 (19.33)	26 (17.33)	
50–59	53 (44.54)	60 (40.00)	
60–69	29 (24.37)	54 (36.00)	
≥ 70	2 (1.68)	1 (0.67)	
Sex			
Female	27 (22.69)	37 (24.67)	0.705
Male	92 (77.31)	113 (75.33)	
ECOG PS			
0	21 (17.65)	32 (21.33)	0.450
1	98 (82.35)	118 (78.67)	
Stage at diagnosis			
II	2 (1.68)	1 (0.67)	0.755
III	7 (5.88)	6 (4.00)	
IV	109 (91.60)	141 (94.00)	
Pathological grading			
Gx	12 (10.53)	21 (15.11)	0.280
G1	6 (5.26)	4 (2.88)	
G2	46 (40.35)	44 (31.65)	
G3	50 (43.86)	70 (50.36)	
No. of metastatic sites			
≤ 2	79 (66.39)	104 (69.80)	0.551
> 2	40 (33.61)	45 (30.20)	
Prior surgery of primary tumor			
Yes	86 (72.27)	108 (72.00)	0.961
No	33 (27.73)	42 (28.00)	
Prior radiotherapy			
Yes	19 (15.97)	21 (14.00)	0.653
No	100 (84.03)	129 (86.00)	
Neoadjuvant chemotherapy			
Yes	32 (26.89)	45 (30.00)	0.575
No	81 (73.11)	105 (70.00)	
Months since diagnosis^c^	15.7 (10.1–29.9)	18.3 (11.8–31.4)	0.1876
Comorbidity			
Yes	29 (24.37)	44 (29.33)	0.363
No	90 (75.63)	106 (70.67)	
Days since last chemotherapy^c^	44 (34–91)	46 (32–91)	0.7310
Tumor size			
≥ 5 cm	50 (42.02)	56 (37.33)	0.435
< 5 cm	69 (57.98)	94 (62.67)	

^a^Adverse events are defined as hypertension, proteinuria, or hand and foot syndrome in the first 4 weeks of treatment

^b^ *P* values were calculated from chi-square or Fisher’s exact test for categorical variables, *t* test for normally distributed continuous variables, and Wilcoxon rank sum test for continuous and skewed variables

^c^Presented as median (interquartile range) ECOG PS: Eastern Cooperative Oncology Group performance status

Throughout the follow-up till 28 days after the last dose of apatinib, no patient had grade 4 (life-threatening or disabling) or grade 5 (death) HTN, proteinuria, or HFS, and only 26 patients (9.67%) developed grade 3 (severe) HTN, proteinuria, or HFS. The AEs were manageable and reversible after treatment interruptions, dose reductions, and supportive care, supporting the tolerability and acceptability of AEs as a biomarker of efficacy.

HTN, proteinuria, and HFS were the three most common AEs and occurred mostly within 4 weeks (cycle 1) after initiation of therapy (Additional file 1: Figure S1). One hundred fifty out of 269 patients (55.76%) treated with apatinib had at least one of the three AEs, HTN, proteinuria, or HFS, in the first four weeks of treatment, of whom 88 patients had only 1, 48 had 2, and only 4 had all three of the AEs. These 150 patients with at least one of the three AEs in the first cycle accounted for 84.7% of patients who had the three AEs during the whole follow-up. The individual AEs were present in 30.86, 29.74, and 23.42% of patients, respectively. The presence of HTN was significantly correlated with proteinuria and HFS (*p* = 0.001 and *p* < 0.001 in chi-square test), but the time of onset of each AE was not associated with the grade of severity or relatedness of the AE with the drug (*p* all > 0.05).

**Fig. 1:**
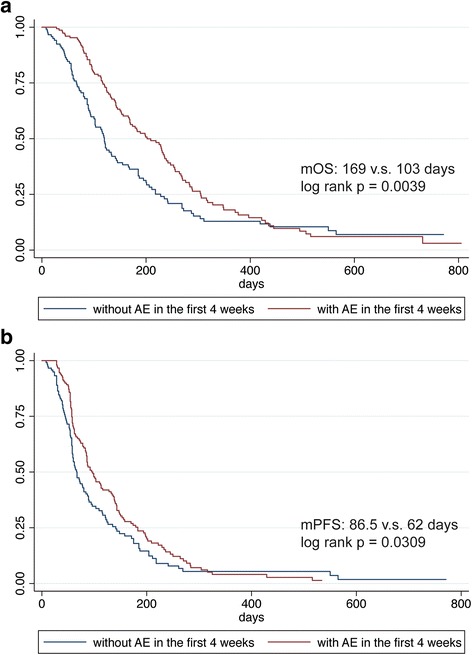
Overall survival and progression-free survival of patients treated with apatinib according to the presence of hypertension, proteinuria, or hand and foot syndrome in the first 4 weeks of treatment. **a** Overall survival. **b** Progression-free survival. mOS median overall survival; mPFS median progression-free survival

The presence of AEs in the first 4 weeks was strongly correlated with better clinical outcomes (Table 2). Patients with vs. without AEs had a median OS of 169 vs. 103 days (log-rank *p* = 0.0039) and a median PFS of 86.5 vs. 62 days (log-rank *p* = 0.0309), respectively (Fig. 1). DCR was significantly higher in patients with AEs (54.67%) compared to those without (32.77%, *p* = 0.0003). ORR was also higher in patients with AEs, but the difference was not statistically significant.

**Table 2:** Correlation between presence of at least one anti-angiogenesis-related adverse event and antitumor efficacy of apatinib

Clinical outcomes	With adverse events (*n* = 150)	Without adverse events (*n* = 119)	Unadjusted analysis		Multi-adjusted analysis^a^	
			HR/OR^b^ (95% CI)	*P* value^c^	HR/OR (95% CI)	*P* value^d^
Median overall survival (IQR), days	169 (96–255)	103 (58–201)	0.67 (0.51,0.88)	0.0039	0.64 (0.48,0.84)	0.001
Median progression-free survival (IQR), days	86.5 (57–150)	62 (41–121)	0.75 (0.58,0.98)	0.0309	0.79 (0.53,0.91)	0.007
Disease control rate, *n* (%)	39 (32.77)	82 (54.67)	2.47 (1.46,4.21)	< 0.001	2.67 (1.59,4.47)	< 0.001
Objective response rate, *n* (%)	6 (5.04)	11 (7.33)	1.49 (0.49.5.06)	0.443	1.42 (0.50,4.01)	0.505

Adverse events are defined as hypertension, proteinuria, or hand and foot syndrome in the first 4 weeks of treatment

*HR* hazard ratio, *OR* odds ratio, *IQR* interquartile range

^a^Adjusted for sex, every 10-year increase in age, number of metastatic sites and ECOG PS

^b^HR for overall survival and progression survival; OR for disease control rate and objective response rate

^c^ *P* values calculated from log-rank test for overall survival and progression survival, and chi-square test for disease control rate and objective response rate

^d^ *P* values calculated from Cox regression for overall survival and progression survival, and logistic regression for disease control rate and objective response rate

The results remained similar after adjusting for potential confounders, including age, sex, ECOG PS, and number of metastatic sites (Table 2). Presence of AEs in the first 4 weeks was associated with a 36% reduction in hazard of death (HR 0.64, 95% CI, 0.48–0.84, *p* = 0.001), a 31% reduction in hazard of progression (HR 0.69, 95% CI, 0.53–0.91, *p* = 0.007), and a 167% increase in DCR (odds ratio (OR) 2.67, 95% CI, 1.59–4.47, *p* < 0.001). Presence of AEs in the 4 weeks stood the proportional hazard assumption, so the effect of the exposure remained constant before and after 400 days (multi-adjusted *p* for interaction term with dichotomous time in Cox regression was 0.081 and > 0.999 for OS and PFS, respectively). The cut-off of 400 days was chosen by visually looking at the log-negative log-survival probability plot (figure not shown). No effect modification was found by age, sex, or ECOG PS (Additional file 2: Figure S2A).

To address potential bias from misclassification of patients who may have not remained on study long enough for AE to be observed, landmark analyses were conducted at the end of 4 weeks (Additional file 3: Table S1). Median OS were statistically prolonged in patients with AE in univariate and multivariate analysis. PFS was not statistically different in crude analysis, but the difference turned significant after adjusting for potential confounders.

The analyses of secondary exposures on primary outcome confirmed the effect of AEs on OS (Additional file 2: Figure S2B). In the first 4 weeks of treatment, the hazard of death decreased by 10% (HR 0.90, 95% CI, 0.83–0.98) for every one week earlier of AE onset, and 23% (HR 0.77, 95% CI, 0.66–0.90) for every one more AE occurred. The onset of any single AE or any combinations of the three AEs in the first 4 weeks were all statistically significantly associated with prolonged OS, except for the presence of proteinuria (HR 0.81, 95% CI, 0.60, 1.09), which showed a same trend but did not reach statistical significance. Different cut-offs of time of AE onset were also explored. The presence of AEs in the first 2 and 3 weeks of treatment also strongly correlated with prolonged OS, while AE in the first week after initiation of treatment failed to meet statistical significance probably because of the limited number of outcomes to reach statistical power.

**Fig. 2:**
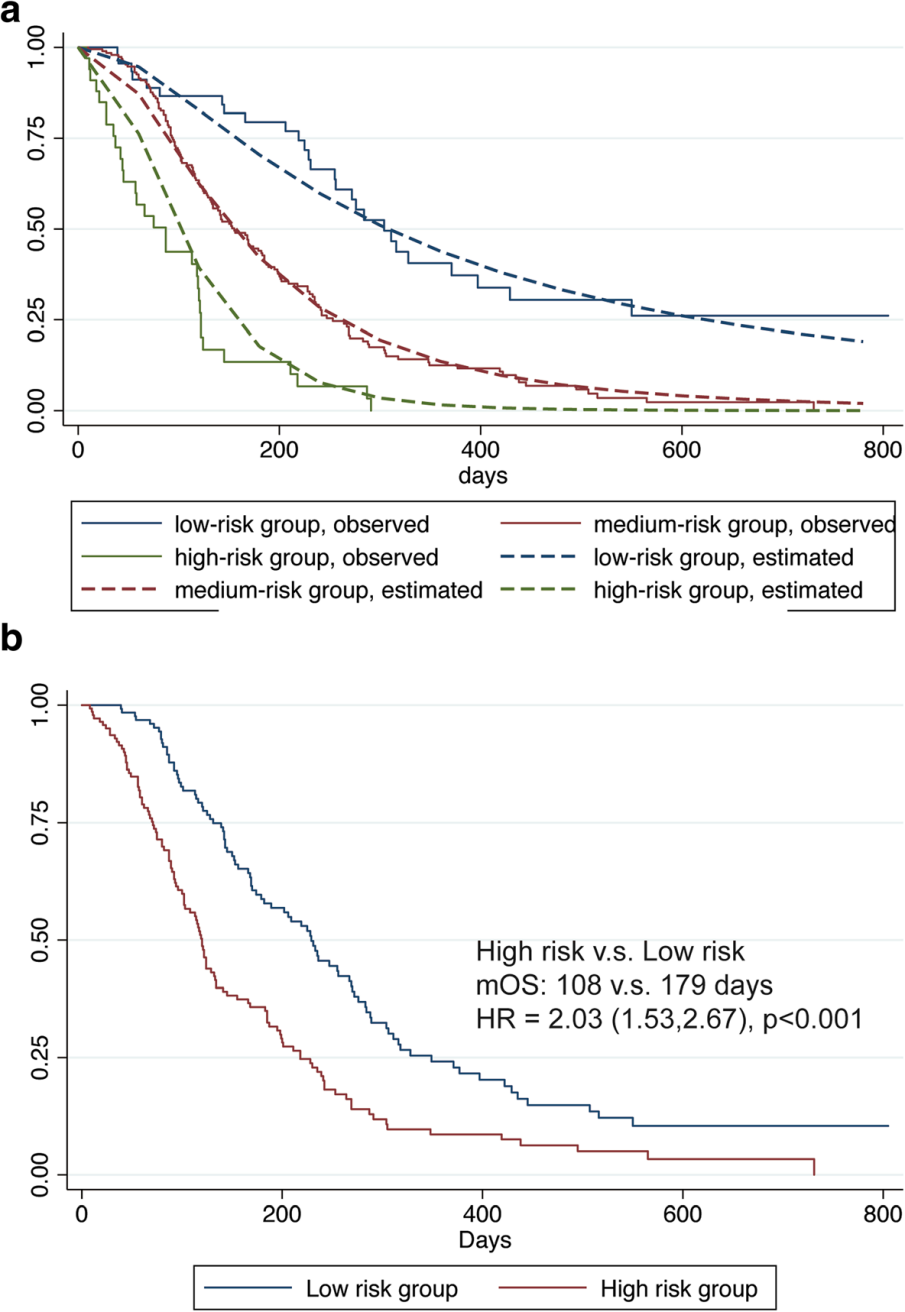
Predictive model and risk score in predicting overall survival. **a** Calibration and discrimination of survival probabilities for the predictive model. **b** Kaplan–Meier curves for overall survival in risk groups according to risk score. mOS median overall survival

A multivariable Cox regression predictive model was constructed to predict OS in the study population. Covariates selected based on subject matter knowledge included absence of AEs in the first 4 weeks, sex, every 10-year increase in age, ECOG PS, more than two metastasis sites, and every 5-cm increase in tumor size. Three covariates, absence of AEs in the first 4 weeks, more than two metastatic sites, and ECOG PS > 0 entered the model after stepwise selection (Table 3). A linear predictor was calculated as a weighted sum of the variables in the model, where the weights were the regression coefficients. The patients were then categorized into three risk groups. Fractional polynomial regression was used to approximate the log baseline cumulative hazard function as previously described [14]. The predicted mean survival curves were compared with the Kaplan–Meier survival curves in the three risk groups to visually assess calibration and discrimination (Fig. 2a). The observed Kaplan–Meier curves in the three risk groups were widely separated, suggesting good discrimination. Calibration was reasonable as the estimated and observed curves in each group were almost identical for all except the high-risk risk group, where the model consistently underpredicted the risk.

**Table 3:** Prediction model and risk score calculation

Risk Factor	HR	95% CI	*P* value	Assigned score
Absence of AE^a^ in first 4 weeks	1.60	(1.22, 2.11)	0.001	2
> 2 metastasis sites	1.62	(1.21, 2.17)	0.001	2
ECOG PS > 0	2.48	(1.70, 3.61)	< 0.001	3

*HR* hazard ratio, *CI* confidence interval, *AE* adverse event, *ECOG PS* Eastern Cooperative Oncology Group performance status

^a^Adverse events (AE) are defined as hypertension, proteinuria, or hand and foot syndrome in the first 4 weeks of treatment

For easier prediction in clinical settings, a point scoring system was applied by assigning 2, 2, and 3 points to the three risk factors in the model, absence of AEs in the first 4 weeks, more than two metastatic sites, and ECOG PS > 0, according to the corresponding HRs in the multivariate model (Table 3). Patients with risk scores of ≤ 5 were assigned as low-risk group, and others were in high-risk group (Additional file 3: Table S2). High-risk patients had more than 2 months shorter OS (107 vs. 179 days, Fig. 2b) and more than 100% higher hazard of death (HR 2.03, 95% CI, 1.53–2.67) compared to low-risk patients. External validation of the model will be conducted after completion of the ongoing phase IV trial.

## Discussion

Several anti-VEGF/VEGFR drugs have been studied in GC. As bevacizumab failed to benefit overall survival in first-line treatment [15], researchers are not optimistic about an anti-VEGF/VEGFR strategy in the first-line setting. As anti-VEGFR-2 drugs, ramucirumab and apatinib were both studied in previously treated GC. The difference between the two drugs lies in two aspects. First, apatinib was given as third-line treatment and ramucirumab was given as second-line therapy. Second, in terms of patient population, both REGARD [5] and RAINBOW [6] studies are worldwide studies that enrolled patients including Caucasians and Asians. However, in the RAINBOW study [6], ramucirumab did not show additional survival benefit in the Asian population. The trials of apatinib were conducted in China. Although the global significance is limited until it has been tested and proven to be effective in other populations, apatinib provides a promising treatment for GC who have failed second-line chemotherapy.

A challenge to the use of apatinib is the need to find biomarkers to predict drug efficacy. In this retrospective cohort analysis, apatinib treatment-induced HTN, proteinuria, and HFS during the first cycle of treatment was associated with statistically significant and clinically meaningful improvement in clinical outcomes, including more than 2-month increase in OS, almost 1-month increase in PFS, and 167% increase in DCR. These findings support the hypothesis that early presence of apatinib treatment-induced AEs is a viable biomarker of antitumor efficacy in metastatic GC patients.

HTN, proteinuria, and HFS are common side effects associated with treatment with angiogenesis inhibitors that target the VEGF pathway, including bevacizumab [16], sorafenib, sunitinib [17], and the novel agent ramucirumab [5]. The mechanisms have not been fully elucidated, but several studies have suggested the inhibition of VEGF pathway in non-tumor cells. Inhibition of VEGFR in vascular endothelial cells decreases the production of nitric oxide and prostacyclins, leading to increased blood pressure [18]. Proteinuria might be induced by inhibition of VEGF in pedal cells and mesangial cells in glomerula [19, 20]. HFS is considered a result of decreased reconstruction of skin after restriction of vessels and has a dose-response relationship with the agents [21]. Similar relationship between angiogenesis inhibitors induced AEs, and treatment efficacy has been identified in various cancers, including renal cell carcinoma, colorectal cancer, and gastrointestinal stromal tumor [17, 22, 23].

AEs induced by angiogenesis inhibitors could partly reflect the inherent host biology that caused the difference in VEGF blockade and thus serve as a biomarker of VEGF pathway inhibition efficacy. Nevertheless, the possibility that the AEs may be independent of VEGF inhibition cannot be excluded. An AE occurs after the initiation of treatment and is not as ideal as the intrinsic biomarkers present before treatment. It has been previously reported that high tumor expression of p-VEGFR2 is an independent prognostic biomarker for prolonged PFS in advanced breast cancer treated with apatinib [10]. However, there is currently no intrinsic biomarker for apatinib in GC. As the AEs presented in this study are manageable, easy to measure, and of low cost and occur early after initiation of therapy, if prospectively validated, they could be a desirable prognostic biomarker for GC patients treated with apatinib.

Several limitations should be considered when interpreting the results. First, full pharmacokinetic data was lacking. It is reasonable to believe that as a target drug, apatinib functions depending more on the efficacy of VEGF pathway blockage, which is mostly explained by individual sensitivity to the drug, than on the concentration of apatinib. In 11 patients from the phase I trial [7], the serum concentration of apatinib at 24 h was not significantly different between patient with and without HTN, as well as HFS (unpublished data). Since all patients received 850 mg per day in total at the beginning, and subsequent dose reduction was an intermediate and thus should not be adjusted for, the current study is valid in showing the association between AEs and clinical outcomes, although pharmacokinetic data could further validate the findings. Second, collinearity may exist among number of metastatic sites, stage, and pathological grade. We only adjusted for number of metastatic sites, since missing values were the least in this variable. In addition, the two trials included in this analysis included only Chinese patients, so it is possible that these results are specific to this patient population only. Whether these results are the same in other population still needs further validation.

## Conclusions

In conclusion, the presence of HTN, proteinuria, or HFS during the first cycle of apatinib treatment correlates with clinical outcomes in GC patients. Prospective studies are warranted in the validation of the presence of these AEs as a biomarker for apatinib antitumor efficacy.

## Additional files

Additional file 1: Figure S1. Manifestation of hypertension, proteinuria, hand and foot syndrome during apatinib treatment. HTN: hypertension; PrU: proteinuria; HFS: hand and foot syndrome. (TIF 1373 kb)

Additional file 2: Figure S2. Subgroup analyses and analyses of secondary exposures. A. Forest plot of subgroup analyses by age, sex, or ECOG PS. B. Forest plot of results from adjusted Cox regression analyses of secondary exposures on overall survival. HTN: hypertension; PrU: proteinuria; HFS: hand and foot syndrome. (TIF 1373 kb)

Additional file 3: Table S1. Landmark analyses. **Table S2.** Distribution of risk scores and risk groups. (DOCX 61 kb)

## Abbreviations

AE: Adverse event
CI: Confidence interval
DCR: Disease control rate
ECOG PS: Eastern Cooperative Oncology Group performance status
GC: Gastric cancer
HFS: Hand and foot syndrome
HR: Hazard ratio
HTN: Hypertension
ORR: Objective response rate
OS: Overall survival
PFS: Progression-free survival
RECIST: Response Evaluation Criteria in Solid Tumors
