# Correction to: Early presence of anti-angiogenesis-related adverse events as a potential biomarker of antitumor efficacy in metastatic gastric cancer patients treated with apatinib: a cohort study

**DOI:** 10.1186/s13045-017-0545-5

**Published:** 2018-01-09

**Authors:** Xinyang Liu, Shukui Qin, Zhichao Wang, Jianming Xu, Jianping Xiong, Yuxian Bai, Zhehai Wang, Yan Yang, Guoping Sun, Liwei Wang, Leizhen Zheng, Nong Xu, Ying Cheng, Weijian Guo, Hao Yu, Tianshu Liu, Pagona Lagiou, Jin Li

**Affiliations:** 1 Department of Epidemiology, Harvard T. H. Chan School of Public Health, 677 Huntington Avenue, Boston, MA 02115 USA; 2 Fudan University Zhongshan Hospital, Shanghai, China; 3 People’s Liberation Army Cancer Center, 81st Hospital of People’s Liberation Army, Beijing, China; 4 Academy of Military Medical Sciences, 307th Hospital of PLA, Beijing, China; 5 First Affiliated Hospital of Nanchang University, Nanchang, China; 6 Harbin Medical University Cancer Hospital, Harbin, China; 7 Shandong Cancer Hospital, Jinan, China; 8 Gansu Cancer Hospital, Lanzhou, China; 9 First Affiliated Hospital of Anhui Medical University, Hefei, China; 10 Shanghai First People’s Hospital, Shanghai, China; 11 XinHua Hospital Affiliated to Shanghai Jiaotong University, Shanghai, China; 12 First Affiliated Hospital of Zhejiang University, Hangzhou, China; 13 Jilin Provincial Cancer Hospital, Changchun, China; 14 Fudan University Shanghai Cancer Center, Shanghai, China; 15 Nanjing Medical University, Nanjing, China; 16 Department of Hygiene, Epidemiology and Medical Statistics, School of Medicine, National and Kapodistrian University of Athens, 75 M. Asias Street, Goudi GR, 115 27 Athens, Greece; 17 Department of Oncology, Shanghai East Hospital, Tongji University School of Medicine, No. 150 Ji Mo Road, Shanghai, 200120 People’s Republic of China

## Correction

The original article [1] contains two errors in Table 2:

1. The data values in the rows ‘Disease control rate’ and ‘Objective response rate’ and the columns ‘With adverse events’ and ‘Without adverse events’ have mistakenly been interchanged between columns; the values ‘39 (32.77)’ and ‘6 (5.04)’ should be swapped with the values ‘82 (54.67)’ and ‘11 (7.33)’ respectively.
2. The value for the row ‘Median progression-free survival (IQR)’ for the ‘HR/OR’ sub-column should be 0.69, not 0.79.

**Table 2:** Correlation between presence of at least one anti-angiogenesis-related adverse event and antitumor efficacy of apatinib

Clinical outcomes	With adverse events (*n* = 150)	Without adverse events (*n* = 119)	Unadjusted analysis		Multi-adjusted analysis^a^	
			HR/OR^b^ (95% CI)	*P* value^c^	HR/OR (95% CI)	*P* value^d^
Median overall survival (IQR), days	169 (96–255)	103 (58–201)	0.67 (0.51,0.88)	0.0039	0.64 (0.48,0.84)	0.001
Median progression-free survival (IQR), days	86.5 (57–150)	62 (41–121)	0.75 (0.58,0.98)	0.0309	0.69 (0.53,0.91)	0.007
Disease control rate, *n* (%)	82 (54.67)	39 (32.77)	2.47 (1.46,4.21)	< 0.001	2.67 (1.59,4.47)	< 0.001
Objective response rate, *n* (%)	11 (7.33)	6 (5.04)	1.49 (0.49.5.06)	0.443	1.42 (0.50,4.01)	0.505

Adverse events are defined as hypertension, proteinuria, or hand and foot syndrome in the first 4 weeks of treatment

*HR* hazard ratio, *OR* odds ratio, *IQR* interquartile range

^a^Adjusted for sex, every 10-year increase in age, number of metastatic sites and ECOG PS

^b^HR for overall survival and progression survival; OR for disease control rate and objective response rate

^c^*P* values calculated from log-rank test for overall survival and progression survival, and chi-square test for disease control rate and objective response rate

^d^*P* values calculated from Cox regression for overall survival and progression survival, and logistic regression for disease control rate and objective response rate

As such, the table displayed ahead shows the correct presentation of Table 2 and should be considered instead.
