# Supplementary figures and images for: Early presence of anti-angiogenesis-related adverse events as a potential biomarker of antitumor efficacy in metastatic gastric cancer patients treated with apatinib: a cohort study
Source: J Hematol Oncol. 2017 Sep 5;10:153. doi: 10.1186/s13045-017-0521-0 (PMC5584332; doi:10.1186/s13045-017-0521-0)

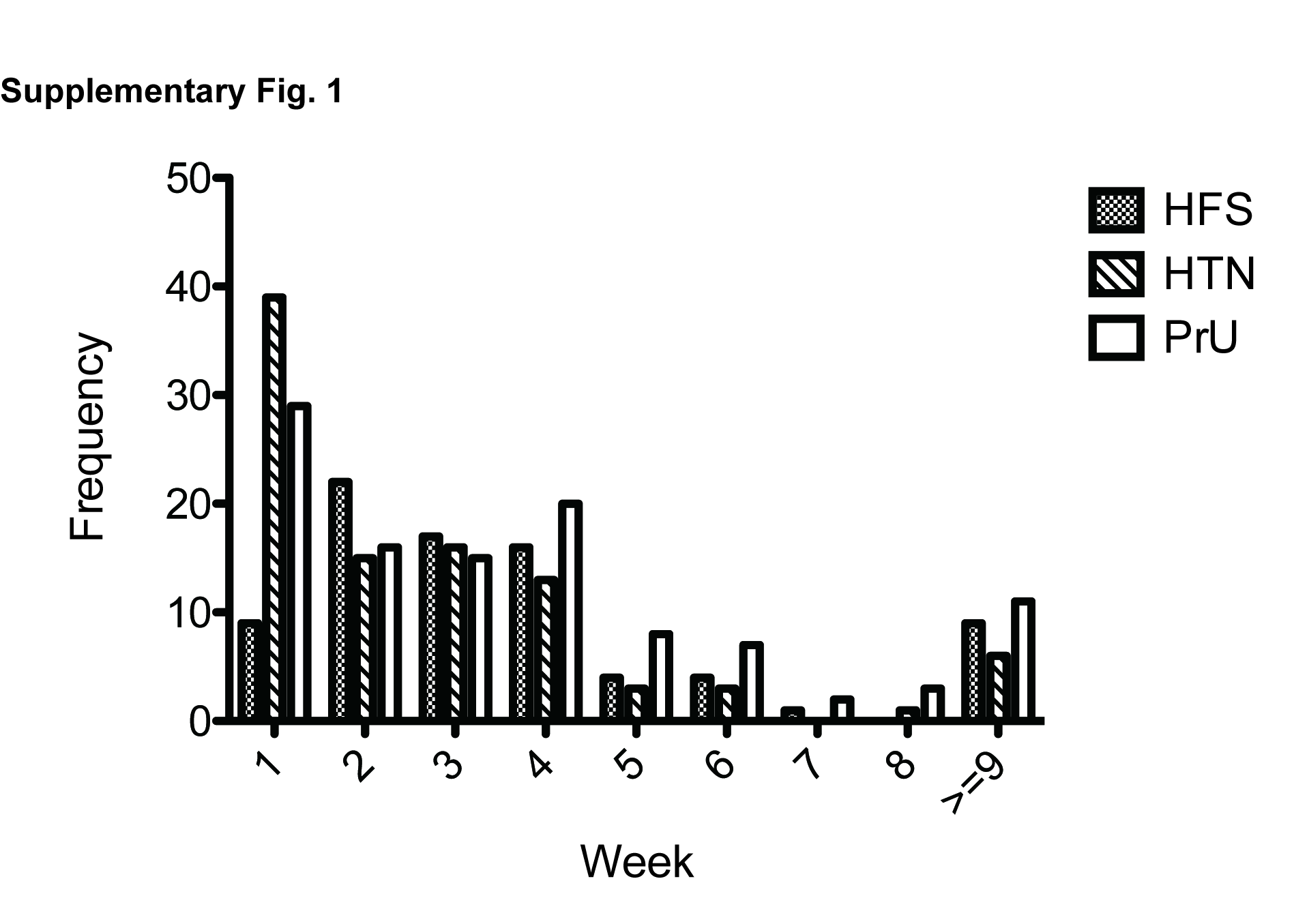

Supplement: Supplementary file 1 — Manifestation of hypertension, proteinuria, hand and foot syndrome during apatinib treatment. HTN: hypertension; PrU: proteinuria; HFS: hand and foot syndrome. (TIF 1373 kb) [file 13045_2017_521_MOESM1_ESM.tif]

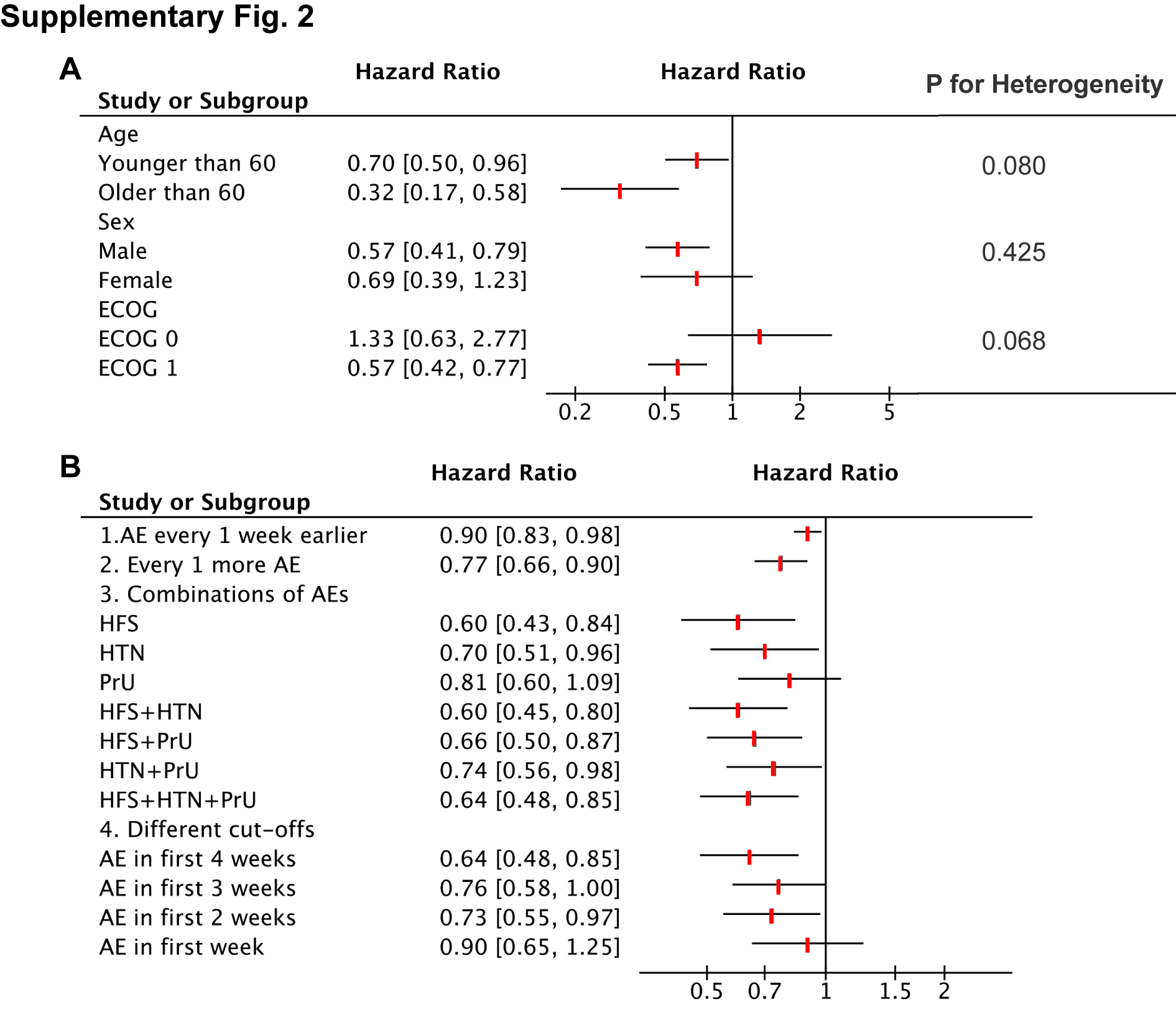

Supplement: Supplementary file 2 — Subgroup analyses and analyses of secondary exposures. A. Forest plot of subgroup analyses by age, sex, or ECOG PS. B. Forest plot of results from adjusted Cox regression analyses of secondary exposures on overall survival. HTN: hypertension; PrU: proteinuria; HFS: hand and foot syndrome. (TIF 1373 kb) [file 13045_2017_521_MOESM2_ESM.tif]
