# Supplementary material for: Early presence of anti-angiogenesis-related adverse events as a potential biomarker of antitumor efficacy in metastatic gastric cancer patients treated with apatinib: a cohort study
Source: J Hematol Oncol. 2017 Sep 5;10:153. doi: 10.1186/s13045-017-0521-0 (PMC5584332; doi:10.1186/s13045-017-0521-0)
Supplement: Supplementary file 3 — Landmark analyses. Table S2. Distribution of risk scores and risk groups. (DOCX 61 kb) [file 13045_2017_521_MOESM3_ESM.docx]

**Table S1. Landmark analyses**

| **Clinical Outcomes** | **With adverse events^a^** | | **Without adverse events** | | **Unadjusted analysis** | | **Multi-adjusted analysis^b^** | |
| --- | --- | --- | --- | --- | --- | --- | --- | --- |
|  | **n** | **Median (IQR), days** | **n** | **Median (IQR), days** | **HR (95% CI)** | **P-value** | **HR (95% CI)** | **P-value** |
| Overall survival | 150 | 169 (96-255) | 112 | 117 (67.5-208) | 0.72 (0.54,0.95) | 0.019 | 0.68 (0.51,0.90) | 0.007 |
| Progression free survival | 150 | 86.5 (57-150) | 109 | 67 (48-124) | 0.81 (0.62,1.06) | 0.121 | 0.74 (0.56,0.97) | 0.031 |

^a^ Adverse events are defined as hypertension, proteinuria, or hand and foot syndrome in the first 4 weeks of treatment.

^b^ Adjusted for sex, every 10-year increase in age, number of metastatic sites and ECOG PS.

IQR: interquartile range; HR: hazard ratio; CI: confidence interval.

**Table S2. Distribution of risk scores and risk groups**

| **Added Score** | **Frequency** | **Percent** | **Group** | **Frequency** | **Percent** |
| --- | --- | --- | --- | --- | --- |
| 2 | 22 | 8.21 | Low risk | 127 | 47.39 |
| 4 | 23 | 8.58 |  |  |  |
| 5 | 82 | 30.6 |  |  |  |
| 6 | 8 | 2.99 | High risk | 141 | 52.61 |
| 7 | 101 | 37.69 |  |  |  |
| 9 | 32 | 11.94 |  |  |  |
